# *Notes from the Field*: Illnesses After Administration of Presumed Counterfeit Botulinum Toxin in Nonmedical Settings — Tennessee and New York City, March 2024

**DOI:** 10.15585/mmwr.mm7327a3

**Published:** 2024-07-11

**Authors:** Christine M. Thomas, Roisin McElroy, Jane Yackley, Mary-Margaret A. Fill, Dilani Goonewardene, Christian Mackley, Emma Roth, Joel Ackelsberg, Sally Slavinski, Caroline Habrun, Bethany Hodge, Carrell Rush, Catherine M. Brown, Michelle A. Waltenburg, Lindsay H. Bertling, Milan McGorty, Renee Johnson, William Schaffner, Timothy F. Jones, John R. Dunn

**Affiliations:** ^1^Epidemic Intelligence Service, CDC; ^2^Tennessee Department of Health; ^3^New York City Department of Health and Mental Hygiene, New York, New York; ^4^Arkansas College of Osteopathic Medicine, Arkansas Colleges of Health Education, Fort Smith, Arkansas; ^5^Kentucky Department for Public Health; ^6^Massachusetts Department of Public Health; ^7^Division of Foodborne, Waterborne, and Environmental Diseases, National Center for Emerging and Zoonotic Infectious Diseases, CDC; ^8^New Orleans District Office, Food and Drug Administration, Nashville, Tennessee; ^9^New York District Office, Food and Drug Administration, New York, New York; ^10^Division of Laboratory Services, Tennessee Department of Health; ^11^Vanderbilt University Medical Center, Nashville, Tennessee.

**Summary**

**What is already known about this topic?**

Administration of botulinum toxin for cosmetic reasons is considered safe in clinical settings, although it can cause transient effects near the injection site.

**What is added by this report?**

During March 2024, seven women experienced illness after receiving botulinum toxin injections in nonmedical settings; four were hospitalized. At least four patients had received counterfeit product.

**What are the implications for public health practice?**

Botulinum toxin injections should be administered by licensed and trained providers using recommended doses of Food and Drug Administration–approved products, preferably in a licensed or accredited health care setting. Clinicians who see patients with suspected botulism should immediately contact their state or local public health department.

Botulinum neurotoxin (BoNT) products are considered safe for cosmetic use when administered in clinical settings, although potential spread of BoNT around the injection site can result in local, transient neurological effects (e.g., ptosis or diplopia) (*1*). In March 2024, clinicians notified the New York City (NYC) Department of Health and Mental Hygiene (DOHMH) and Tennessee Department of Health (TDH) of illnesses after presumed cosmetic BoNT injections. A multistate investigation, which included the Food and Drug Administration (FDA) and CDC, sought to characterize these illnesses and identify implicated BoNT products.

## Investigation and Outcomes

Health department staff members interviewed patients and reviewed medical records to obtain information about patients’ signs and symptoms, health care encounters, and exposure to BoNT products. Product information was shared with FDA. TDH Division of Laboratory Services tested patient specimens for BoNT.* This activity was reviewed by CDC, deemed not research, and conducted consistent with applicable federal law and CDC policy.^†^

NYC DOHMH identified three patients, and TDH identified four (including one Kentucky resident who was admitted to a Tennessee hospital). All patients were women, aged 26–55 years (median age = 48 years). Reported signs and symptoms included ptosis, dry mouth, dysphagia, shortness of breath, and weakness (Table), with onset during February 23–March 7, 2024. All patients sought health care for their illness; four were hospitalized, and two were monitored in intensive care units. None required intubation. CDC’s Botulism Consultation Service determined that botulinum antitoxin was not indicated for any of the seven patients.^§^ All patients reported receiving cosmetic BoNT injections in nonmedical settings a median of 3 days (range = 2–20 days) before symptom onset. Serum and stool specimens collected from two patients approximately 3 weeks after symptom onset tested negative for BoNT, likely because of the interval between symptom onset and specimen collection.

**TABLE:** Characteristics of illnesses after administration of presumed counterfeit botulinum toxin in nonmedical settings — Tennessee and New York City, February–March 2024

Characteristic	No. (column %)		
	Tennessee n = 4	New York City n = 3	Total N = 7
**Age, yrs, median (range)**	43 (39–48)	51 (26–55)	**48 (26–55)**
**Sex**			
Female	4 (100)	3 (100)	**7 (100)**
First sign or symptom*			
Ptosis	4 (100)	1 (33)	**5 (71)**
Diplopia	1 (100)	2 (67)	**3 (43)**
Headache	2 (50)	0 (—)	**2 (28)**
Weakness	2 (50)	0 (—)	**2 (28)**
Blurred vision	0 (—)	1 (33)	**1 (14)**
Signs and symptoms*			
Ptosis	4 (100)	3 (100)	**7 (100)**
Dry mouth	4 (100)	3 (100)	**7 (100)**
Dysphagia	4 (100)	3 (100)	**7 (100)**
Shortness of breath	4 (100)	3 (100)	**7 (100)**
Weakness	4 (100)	3 (100)	**7 (100)**
Blurred vision	4 (100)	2 (67)	**6 (86)**
Diplopia	3 (75)	3 (100)	**6 (86)**
Change in voice or hoarseness	4 (100)	2 (67)	**6 (86)**
Paresthesia	4 (100)	2 (67)	**6 (86)**
Fatigue	4 (100)	0 (—)	**4 (57)**
Nausea	3 (75)	0 (—)	**3 (43)**
Vomiting	2 (50)	0 (—)	**2 (29)**
Urinary retention or incontinence	2 (50)	0 (—)	**2 (29)**
Drooling or pooling of secretions	0 (—)	2 (67)	**2 (29)**
Thick tongue	1 (25)	0 (—)	**1 (14)**
Slurred speech	1 (25)	0 (—)	**1 (14)**
Health care encounter*	4 (100)	3 (100)	**7 (100)**
Admitted to a hospital	2 (50)	2 (67)	**4 (57)**
Admitted to intensive care unit	1 (25)	1 (33)	**2 (29)**
Mechanical ventilation	0 (—)	0 (—)	**0 (—)**
Injection site*			
Face (e.g., forehead or glabella)	4 (100)	3 (100)	**7 (100)**
Neck	0 (—)	2 (67)	**3 (43)**
Trapezius	0 (—)	1 (33)	**1 (14)**
Axillae	0 (—)	1 (33)	**1 (14)**
**Injection setting**			
Residence	4 (100)	2 (67)	**6 (86)**
Cosmetic spa	0 (—)	1 (33)	**1 (14)**

* Some persons reported multiple signs or symptoms, health care encounter types, or injection sites.

The three Tennessee residents and the Kentucky resident received injections of presumed BoNT in a nonmedical residential setting from a relative of one of the recipients, who was not licensed to administer these injections. FDA determined that the BoNT product administered to those four persons was counterfeit.^¶^ The three NYC patients had no epidemiologic links to one another or to the Tennessee and Kentucky patients. The three NYC residents also received injections of presumed BoNT in separate nonmedical settings, with administration by an unlicensed person confirmed for one resident and suspected for two. Product information was not available; however, one person reported paying less than U.S. wholesale acquisition cost for the administered product, and another reported that the product had been purchased overseas.

## Preliminary Conclusions and Actions

Seven persons experienced illness consistent with local and possible distant spread of BoNT after injection of presumed counterfeit BoNT product by unlicensed persons in nonmedical settings. Severe and potentially fatal illnesses associated with unlicensed product and off-label BoNT use have been reported (*2*,*3*). This investigation did not determine why these illnesses occurred after cosmetic BoNT injections; potential reasons might include use of counterfeit BoNT, which might be more potent or contain harmful additional ingredients or higher susceptibility to BoNT effects among some persons. Further studies are needed to describe the clinical spectrum of cosmetic BoNT injection effects (e.g., severity of signs and symptoms).

Health care providers should ask patients with symptoms of botulism about recent BoNT injections and, if botulism is suspected, immediately contact their local or state health departments.** Health departments should investigate reports of possible botulism and, if indicated, consult CDC regarding antitoxin release and notify other federal agencies to identify and remove counterfeit BoNT products from the market. BoNT injections should be administered only by licensed and trained providers using recommended doses of FDA-approved products.

## Footnotes

* Patient stool was tested for *Clostridium botulinum* toxin genes A–G by polymerase chain reaction and, if test results were negative, testing was repeated 5 days later after culture enrichment. Patient serum was tested using mouse bioassay.

† 45 C.F.R. part 46, 21 C.F.R. part 56; 42 U.S.C. Sect. 241(d); 5 U.S.C. Sect. 552a; 44 U.S.C. Sect. 3501 et seq.

§ CDC’s Botulism Consultation Service provides consultation for health departments and clinicians and releases botulinum antitoxin when indicated. For the cases described in this report, antitoxin was not released because some patients’ signs and symptoms were consistent with transient effects of toxin, or signs of neurologic injury were not ongoing or progressing. In addition, >10 days had passed for all patients since onset of symptoms, at which point antitoxin would offer minimal benefit, because botulinum toxin was unlikely to be circulating in the blood.

¶ The person who administered the presumed botulinum toxin product provided product photographs. After communication with the manufacturer, FDA determined the product was counterfeit. Packaging claimed to contain 150 units of “Botulinum Toxin Type A” (brand name “Botox”) manufactured by a company that only manufactures 50-unit, 100-unit, and 200-unit vials of Botox. Manufacturing location in Ireland was misspelled on the packaging. The batch number on the vial label (C3709C3) belonged to a legitimate 100-unit strength batch that expired in August 2017.

** Symptoms of botulism can include ptosis, blurred and double vision, voice changes, dry mouth, drooling or pooling of secretions, dysphagia, shortness of breath, muscle weakness, and fatigue. The most severe signs of botulism include descending paralysis and respiratory failure.

## References

[1] Witmanowski H, Błochowiak K. The whole truth about botulinum toxin—a review. Postepy Dermatol Alergol 2020;37:853–61. 10.5114/ada.2019.82795 33603602 PMC7874868

[2] Chertow DS, Tan ET, Maslanka SE, Botulism in 4 adults following cosmetic injections with an unlicensed, highly concentrated botulinum preparation. JAMA 2006;296:2476–9. 10.1001/jama.296.20.2476 17119144

[3] Dorner MB, Wilking H, Skiba M, A large travel-associated outbreak of iatrogenic botulism in four European countries following intragastric botulinum neurotoxin injections for weight reduction, Türkiye, February to March 2023. Euro Surveill 2023;28:2300203. 10.2807/1560-7917.ES.2023.28.23.2300203 37289431 PMC10318948

